# Stage-specific impact of portal hypertension on outcomes after liver resection in hepatocellular carcinoma

**DOI:** 10.1007/s12072-025-10879-3

**Published:** 2025-07-30

**Authors:** Yukihiro Watanabe, Masayasu Aikawa, Yoshiki Murase, Kenichiro Takase, Yuichiro Watanabe, Hiroaki Ono, Katsuya Okada, Kojun Okamoto, Isamu Koyama

**Affiliations:** Department of Gastroenterological Surgery, Saitama Medical University International Medical Center, 1397-1 Yamane, Hidaka, Saitama 350-1298 Japan

**Keywords:** Clinically significant portal hypertension, Cohort study, Japan integrated staging (JIS) score, Stage-based analysis, Overall survival, Postoperative outcomes, Minimally invasive surgery, Prognostic factor, Cirrhosis, Liver decompensation

## Abstract

**Background and aims:**

The impact of clinically significant portal hypertension (CSPH) on patients with hepatocellular carcinoma (HCC) after liver resection remains controversial. This study evaluated the effect of CSPH on postoperative outcomes in very-early, early, and intermediate-stage HCC.

**Methods:**

In total, 857 patients with HCC undergoing liver resection were identified from a single-institution database. Patients were stratified using the Japan integrated staging (JIS) score, which combines Child–Pugh grade (A: 0, B: 1, C: 2) and TNM stage (I: 0, II: 1, III: 2, IV: 3). Patients were categorized as very-early (JIS 0, *n* = 198), early (JIS 1, *n* = 272), and intermediate (JIS 2–4, *n* = 287). Multivariable Cox regression assessed overall survival (OS).

**Results:**

Risk-adjusted hazard ratios [95% confidence intervals] for OS in CSPH versus non-CSPH patients were 2.18 [1.14–4.18] (very-early), 0.67 [0.40–1.11] (early), and 1.67 [1.10–2.56] (intermediate). In early stage HCC, CSPH had no significant impact on OS, liver failure (19% vs. 19%, *p* = 0.925), 90-day mortality (2.6% vs. 2.9%, *p* = 1.000), or liver decompensation-related death (12% vs. 15%, *p* = 0.758). However, CSPH patients in very-early and intermediate stages had significantly higher liver failure (0.7% vs. 6.9%, *p* = 0.027; 6.0% vs. 19%, *p* = 0.001) and decompensation-related deaths (3.3% vs. 18%, *p* = 0.027; 9.0% vs. 34%, *p* < 0.001).

**Conclusions:**

CSPH’s impact varied by stage. In early stage HCC, CSPH had minimal effect on survival. However, in very-early and intermediate stages, CSPH was associated with worse outcomes. Refining surgical criteria for patients who have HCC with CSPH is essential to balance oncological benefits and hepatic risks.

**Graphical abstract:**

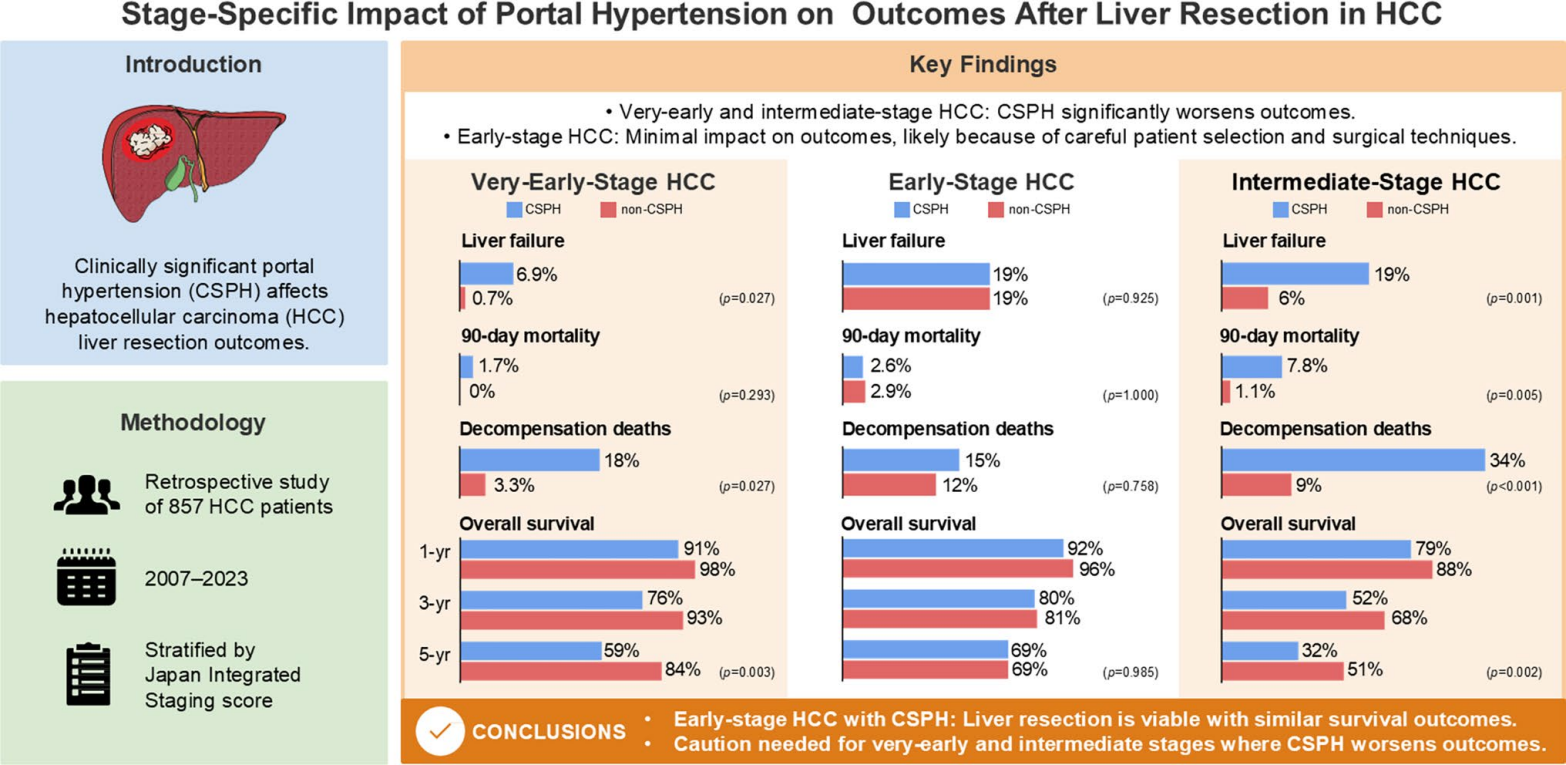

**Supplementary Information:**

The online version contains supplementary material available at 10.1007/s12072-025-10879-3.

## Introduction

Hepatocellular carcinoma (HCC) is the seventh most common cancer and the third leading cause of cancer-related death worldwide [1]. Patients with HCC frequently have concomitant clinically significant portal hypertension (CSPH), which manifests as thrombocytopenia caused by splenomegaly and esophagogastric varices. Because CSPH is a known risk factor for morbidity and mortality after liver resection (LR), its presence has been considered a contraindication to LR, especially in Western countries [2, 3]. Consequently, patients with HCC and CSPH generally have poorer survival than those without CSPH.

Liver transplantation has been regarded as an ideal treatment option for those patients [4]. However, donor shortages and strict criteria limit transplantation eligibility [5]. The Barcelona Clinic Liver Cancer (BCLC) staging system in 2022 suggested that LR can be an option for patients with HCC who have mild CSPH, expanding the surgical indications under specific conditions [6].

Previous studies on CSPH and surgical outcomes have reported conflicting findings. While many studies reported poor outcomes for patients with CSPH [2, 7, 8], some studies indicated no significant difference [9, 10]. These discrepancies may be explained by the heterogeneity of patient backgrounds, tumor characteristics, and liver function, all of which affect overall survival (OS). Therefore, a more rigorous and comprehensive review of the effect of CSPH is needed to ensure safe and effective surgery.

Prognostic staging systems based on liver function and tumor factors, such as the Japan Integrated Staging score (JIS-score), have been adopted in practice and are well validated [11, 12]. These models facilitate comparisons among patients with similar prognostic risks and may more accurately capture the impact of CSPH on surgical outcomes [12]. However, a few studies have systematically stratified patients by these validated models to assess CSPH’s stage-specific impact.

This study aimed to refine surgical decision-making by evaluating postoperative outcomes in patients with HCC and CSPH, stratified by the JIS-score. Using a single-center database in Japan, we assessed the impact of CSPH across various prognostic stages.

## Patients and methods

### Patient selection and study design

This single-center, retrospective cohort study analyzed a prospectively maintained database of LR procedures performed at Saitama Medical University International Medical Center from January 2007 to December 2023. All cases were diagnosed based on computed tomography or magnetic resonance imaging findings, without preoperative biopsy.

### Inclusion and exclusion criteria

Inclusion criteria:

- Age of ≥ 18 years
- Anatomical or non-anatomical hepatectomy
- Curative-intent surgery for primary HCC
- Up to one prior locoregional therapy (e.g., ablation or transcatheter arterial chemoembolization).

Exclusion criteria:

- Metastatic disease
- Exploratory surgery
- Major vascular invasion requiring reconstruction (main portal vein, hepatic artery, biliary duct, or inferior vena cava)
- Extrahepatic resection.

### Data collection and variables

Clinical and pathological data including:

- Demographic information: age, sex, body mass index, American Society of Anesthesiologists performance score, and etiology
- Comorbidities: hypertension, hyperglycemia, dyslipidemia, ischemic heart disease, and chronic renal disease
- Liver function assessments: Child–Pugh grade, Model for End-Stage Liver Disease (MELD) score, albumin–bilirubin (ALBI) grade, ascites, and varices
- Oncologic factors: tumor–node–metastasis (TNM) classification (Union for International Cancer Control, 8th edition), previous treatment history, tumor size and grade, microvascular invasion, capsular invasion, and margin status
- Surgical variables: resection type (anatomical or non-anatomical), surgical approach (open or minimally invasive [laparoscopic or robotic]), intraoperative blood loss, transfusions, and operative time
- Postoperative outcomes: 90-day morbidity, complications, major morbidities (graded using the Clavien–Dindo classification) [13], postoperative liver failure [14], length of hospital stay, recurrence-free survival (RFS), and OS.

Patient data, including complications, were obtained from electronic medical records. This study adhered to the Declaration of Helsinki and was approved by the Institutional Review Board (No. 2024-121). Informed consent was obtained from all patients or waived by the IRB because of the retrospective design. The study followed the STROBE guidelines [15].

### Surgical indication

Treatment strategies for HCC followed the Japan Society of Hepatology algorithm [16]. All patients were evaluated preoperatively by a multidisciplinary team. Indications for preoperative endoscopic treatment of esophageal varices included moderately enlarged, beady varices (F2) or markedly enlarged, nodular or tumor-shaped varices (F3) with a red color sign. Surgical eligibility was determined primarily by liver function (Child–Pugh grade, MELD score, and ALBI score), with preoperative three-dimensional liver reconstruction ensuring ≥ 40% residual liver volume. Tumor number and CSPH were not considered absolute contraindications.

### CSPH

Hepatic venous pressure gradient (HVPG) measurement was not routinely performed because of its invasive nature and limited availability. Instead, validated surrogate markers were used [17, 18]. Patients were classified as having CSPH if they met either of the following criteria:

- Endoscopic evidence of esophageal or gastric varices
- Presence of significant splenomegaly (spleen diameter of > 12 cm) combined with thrombocytopenia (platelet count of < 100,000/mm.^3^).

### Prognostic staging system

The JIS-score, based on the Child–Pugh score and the TNM system developed by the Japan Liver Cancer Study Group (Table 1) [12], has been validated as a prognostic tool in Japan and other Asian countries. Patients were classified into three groups:

- Very-early stage: JIS-score 0
- Early stage: JIS-score 1
- Intermediate stage: JIS-score ≥ 2.

**Table 1:** Japan integrated staging (JIS) score system

Variables	Score 0	Score 1	Score 2	Score 3
Child–Pugh grade	A	B	C	—
TNM stage by LCSGJ	I	II	III	IV
Stage definitions by the liver cancer study group of Japan (LCSGJ)				
Stage I: T1 (fulfilling 3 T factors) N0M0				
Stage II: T2 (fulfilling 2 T factors) N0M0				
Stage III: T3 (fulfilling 1 T factor) N0M0				
Stage IV: T4 (fulfilling 0 T factor) N0M0 or any T N0-1M1				
T factor criteria				
(1) Single tumor				
(2) Tumor size < 2 cm				
(3) No vascular involvement				

### Outcomes

The primary clinical outcome was OS, defined as the time from surgery to all-cause death. Follow-up was conducted every 3 months for 3 years, and then every 6 months. Those without the event were censored at the final follow-up.

Secondary outcomes included RFS, postoperative complications (Clavien–Dindo classification), and 90-day mortality.

### Definitions

- Liver decompensation: Development of ascites, hepatic encephalopathy, variceal bleeding, or jaundice.
- Metabolic syndrome: Defined according to established criteria as a combination of central obesity, insulin resistance, hypertension, dyslipidemia, and hyperglycemia [19].
- Esophagogastric varices: low-risk group (F1–F2 without red color sign) and high-risk group (F2–F3 with red color sign or history of prior endoscopic treatment)
- Tumor characteristics: Tumor size, number, differentiation, microvascular invasion, capsular invasion, cirrhosis (pathologically confirmed), and R0 resection status were determined according to the General Rules for the Clinical and Pathological Study of Primary Liver Cancer [20].
- Complication severity: Graded using the Clavien–Dindo classification (Grade I–II: minor; Grade ≥ III: major) [13].
- Microvascular invasion: Defined as the presence of tumor emboli within the central vein, portal vein, large capsular vessels, or involvement of segmental or sectional branches of the portal or hepatic veins.

### Statistical analysis

Continuous data are presented as median (interquartile range), and categorical data as number (percentage). The Chi-square test or Fisher’s exact test was used to analyze categorical variables. OS was estimated using the Kaplan–Meier method and compared with the log-rank test. Univariate and multivariate analyses (Cox regression) identified prognostic factors, with variables selected based on clinical relevance, prior studies, and sample size (to avoid overfitting for very-early stage HCC).

A *p* value of < 0.05 was considered statistically significant. All analyses were performed using STATA, version 15.0 (StataCorp, College Station, TX, US).

## Results

Table 2 presents the patients’ characteristics stratified by JIS-score staging. In very-early stage HCC, the CSPH group was younger. As the JIS-score stage increased, the proportion of high-risk varices in the CSPH group also increased. Although ALBI grade and Child–Pugh grade showed no significant differences, the platelet count, bilirubin concentration, and prothrombin time were worse in the CSPH group, and the alpha-fetoprotein (AFP) levels were higher despite similar tumor sizes. In early stage HCC, the CSPH group had a higher prevalence of hepatitis C and worse liver function markers (Child–Pugh grade, ALBI score, MELD score, prothrombin time, albumin concentration, and bilirubin concentration) as well as lower platelet count. Tumor-related factors, such as tumor size and protein induced by vitamin k absence or antagonist-II levels, were more favorable in the CSPH group, whereas AFP concentration, tumor differentiation, and microvascular invasion were comparable. In intermediate-stage HCC, significant differences were observed in age, sex, American Society of Anesthesiologists score, cancer stage, platelet count, AFP levels, microvascular invasion, and liver function-related variables. Additionally, non-anatomical resection and minimally invasive surgery (MIS) were performed more frequently in patients with early and intermediate-stage CSPH.

**Table 2:** Characteristics in patients stratified according to JIS-score

Stage	Very-early stage (JIS-score 0)			Early stage (JIS-score 1)			Intermediate stage (JIS-score 2–4)		
Characteristic	Non-CSPH (*n* = 140)	CSPH (*n* = 58)	*p*	Non-CSPH (*n* = 267)	CSPH (*n* = 105)	*p*	Non-CSPH (*n* = 184)	CSPH (*n* = 103)	*p*
Age, years	73 (67–77)	69 (64–74)	0.006*	72 (66–77)	70 (65–76)	0.259	71 (66–76)	70 (64–73)	0.040*
Sex, male	106 (76)	38 (66)	0.143	210 (79)	73 (70)	0.063	154 (84)	71 (69)	0.004*
BMI, kg/m^2^	23.2 ± 3.6	23.4 ± 4.4	0.838	23.7 ± 3.5	23.5 ± 3.5	0.591	23.8 ± 3.3	23.8 ± 3.9	0.957
ASA score			0.210			0.061			0.002*
≤ 2	121 (86)	46 (79)		236 (88)	85 (81)		159 (86)	74 (72)	
> 2	19 (14)	12 (21)		31 (12)	20 (19)		25 (14)	29 (28)	
Etiology			0.039*			< 0.001*			< 0.001*
Hepatitis C	77 (55)	35 (60)		104 (39)	61 (58)		76 (41)	63 (61)	
Hepatitis B	13 (9.3)	2 (3.5)		26 (9.7)	5 (4.8)		15 (8.1)	10 (9.7)	
NAFLD/NASH	35 (25)	8 (14)		105 (39)	18 (17)		67 (36)	14 (14)	
Alcohol/AIH	15 (11)	13 (22)		32 (12)	21 (20)		26 (14)	16 (16)	
Metabolic syndrome	101 (72)	37 (64)	0.245	204 (76)	84 (80)	0.455	142 (77)	70 (68)	0.088
Central obesity	37 (26)	12 (21)	0.394	89 (33)	34 (32)	0.860	70 (38)	34 (33)	0.395
Hyperglycemia	44 (31)	22 (38)	0.410	99 (37)	44 (42)	0.409	73 (40)	34 (33)	0.309
Hypertension	69 (49)	20 (35)	0.057	148 (55)	51 (49)	0.233	97 (53)	47 (46)	0.249
Dyslipidemia	9 (6.4)	1 (1.7)	0.286	24 (9.0)	4 (3.8)	0.125	13 (7.1)	4 (3.9)	0.311
Ischemic heart disease	6 (4.3)	1 (1.7)	0.676	22 (8.2)	4 (3.8)	0.175	15 (8.2)	8 (7.8)	1.000
Chronic renal failure	9 (6.4)	1 (1.7)	0.286	22 (8.2)	8 (7.6)	1.000	10 (5.4)	7 (6.8)	0.614
Cancer stage			1.000			0.917			< 0.001*
IA–IB	140 (100)	58 (100)		245 (91)	96 (91)		7 (3.8)	37 (36)	
II–IIIB	0	0		22 (8.2)	8 (8.6)		177 (96)	66 (64)	
Child–Pugh grade			1.000			< 0.001*			< 0.001*
A	140 (100)	58 (100)		261 (98)	80 (76)		159 (86)	36 (35)	
B	0	0		6 (2.3)	25 (24)		25 (14)	67 (65)	
MELD score	7.24 (6.43–7.99)	7.95 (7.29–8.47)	< 0.001*	7.19 (6.43–8.41)	8.19 (7.29–9.23)	< 0.001*	7.08 (6.43–8.47)	8.75 (7.60–10.2)	< 0.001*
ALBI score			0.194			< 0.001*			< 0.001*
Grade 1	94 (67)	33 (57)		181 (68)	48 (46)		110 (60)	17 (17)	
Grade 2	46 (33)	25 (43)		86 (32)	55 (52)		74 (40)	81 (80)	
Grade 3	0	0		0	2 (1.9)		0	4 (3.9)	
Varices			< 0.001*			< 0.001*			< 0.001*
None	140 (100)	3 (8.6)		267 (100)	7 (6.6)		184 (100)	5 (4.9)	
F1–F2 without RC sign	0	45 (78)		0	76 (72)		0	67 (65)	
F2–F3 with RC sign or prior EVL (EIS)	0	10 (17)		0	22 (21)		0	31 (30)	
Pre-locoregional therapy	29 (21)	18 (31)	0.120	45 (17)	24 (23)	0.180	33 (18)	24 (23)	0.274
Platelet count, × 10^3^/μL	167 ± 52	88 ± 27	< 0.001*	182 ± 57	95 ± 39	< 0.001*	190 ± 82	90 ± 49	< 0.001*
Albumin, g/dL	4.0 (3.7–4.3)	3.9 (3.7–4.2)	0.293	4.1 (3.7–4.3)	3.8 (3.5–4.2)	< 0.001*	3.9 (3.6–4.2)	3.4 (3.1–3.8)	< 0.001*
Bilirubin, mg/dL	0.6 (0.5–0.8)	0.7 (0.6–1.0)	< 0.001*	0.6 (0.5–0.8)	0.8 (0.5–1.1)	< 0.001*	0.6 (0.5–0.8)	0.8 (0.6–1.3)	< 0.001*
PT, %	92 (84–100)	79 (74–89)	< 0.001*	93 (84–102)	80 (71–89)	< 0.001*	91 (80–100)	74 (64–86)	< 0.001*
AFP, ng/mL	5.2 (3.4–13)	13 (5.4–52)	< 0.001*	6.6 (3.1–23)	9.7 (4.3–34)	0.015*	9.5 (4.4–138)	28 (6.2–236)	0.013*
PIVKA-II, mAU/mL	27 (20–45)	23 (18–53)	0.192	59 (24–342)	33 (21–92)	0.001*	121 (36–1522)	139 (38–629)	0.490
Neutrophil-to-lymphocyte ratio	2.0 (1.4–2.7)	2.2 (1.3–2.7)	0.705	2.1 (1.5–2.9)	2.1 (1.5–2.9)	0.705	2.4 (1.6–3.4)	2.2 (1.5–3.2)	0.182
Lesion location			0.755			0.814			0.154
Anterolateral	75 (55)	29 (50)		117 (44)	44 (42)		57 (31)	43 (42)	
Posterosuperior	65 (46)	29 (50)		132 (49)	52 (50)		70 (38)	36 (35)	
Multiple	0	0		18 (6.7)	9 (8.6)		57 (31)	24 (23)	
Resection type			0.623			< 0.001*			< 0.001*
Non-anatomical	126 (90)	51 (88)		150 (56)	86 (82)		75 (41)	72 (70)	
Anatomical	14 (10)	7 (12)		117 (44)	19 (18)		109 (59)	31 (30)	
R1 resection	6 (4.3)	2 (3.6)	1.000	10 (3.8)	8 (7.6)	0.117	15 (8.1)	10 (9.7)	0.654
Tumor size, cm	1.5 (1.2–1.8)	1.5 (1.2–1.7)	0.237	3.2 (2.3–5.0)	2.3 (1.7–3.0)	< 0.001*	5.0 (3.0–8.0)	2.8 (2.3–4.7)	< 0.001*
Differentiation			0.744			0.312			0.754
Well or moderate	132 (94)	51 (93)		246 (92)	93 (89)		151 (82)	83 (81)	
Poor	8 (5.8)	4 (6.9)		21 (7.9)	12 (11)		33 (18)	20 (19)	
Microvascular invasion	0	0		16 (6.0)	9 (8.6)	0.365	129 (70)	51 (50)	0.001*
Capsular invasion	46 (33)	18 (32)	0.868	100 (38)	41 (39)	0.813	83 (45)	45 (45)	1.000
Cirrhosis	67 (48)	54 (93)	< 0.001*	91 (34)	98 (94)	< 0.001*	77 (42)	94 (92)	< 0.001*
Liver resection			0.240			< 0.001*			0.002*
Open surgery	46 (33)	14 (25)		147 (55)	36 (34)		118 (64)	46 (45)	
Minimum invasive surgery	94 (67)	44 (75)		120 (45)	69 (66)		66 (36)	57 (55)	

Data are presented as *n*, *n* (%), or median (interquartile range)

JIS, Japan Integrated Staging; NAFLD, non-alcoholic fatty liver disease; BMI, body mass index; ASA, American Society of Anesthesiologists; MELD, Model for End-stage Liver Disease; RC sign, red color sign; EVL, endoscopic variceal ligation; EIS, endoscopic injection sclerotherapy; PT, prothrombin time; AFP, alpha-fetoprotein; PIVKA-II, protein induced by vitamin K absence-II

*Statistically significant

Table 3 presents the perioperative outcomes. In very-early stage HCC, the CSPH group had a significantly higher incidence of transfusion and postoperative liver failure than the non-CSPH group, but no significant differences were observed in surgical duration, blood loss, 90-day mortality, major morbidity, complications, or postoperative stay. In early and intermediate-stage HCC, the CSPH group had significantly higher transfusion rates but shorter surgical duration, less blood loss, and shorter postoperative stays, while major morbidity and overall complication rates remained comparable between the two groups. However, in intermediate-stage HCC, the CSPH group exhibited significantly higher 90-day mortality and postoperative liver failure rates, whereas these outcomes did not differ significantly in early stage HCC. Regarding oncological outcomes, RFS was comparable between the two groups across all stages, but OS was significantly worse in the CSPH group for very-early and intermediate-stage HCC, while remaining comparable in early stage HCC.

**Table 3:** Perioperative and survival outcomes in patients stratified according to JIS-score

Characteristic	Very-early stage (JIS-score 0)			Early stage (JIS-score 1)			Intermediate stage (JIS-score 2–4)		
	Non-CSPH (*n* = 140)	CSPH (*n* = 58)	*P*	non-CSPH (*n* = 267)	CSPH (*n* = 105)	*p*	non-CSPH (*n* = 184)	CSPH (*n* = 103)	*p*
	Non-CSPH	CSPH		non-CSPH	CSPH		non-CSPH	CSPH	
Surgical duration, minutes	148 (106–198)	167 (117–209)	0.101	203 (141–274)	144 (121–218)	< 0.001*	230 (169–306)	195 (129–249)	0.001*
Blood loss, mL	50 (5–185)	50 (5–140)	0.581	188 (50–575)	50 (5–250)	< 0.001*	374 (104–911)	200 (5–660)	0.010*
Duration of Pringle maneuver, minutes	12 (0–22)	15 (5–25)	0.782	32 (10–43)	28 (12–38)	0.681	42 (16–48)	35 (15–41)	0.386
Transfusion	4 (2.9)	15 (26)	< 0.001*	47 (18)	29 (28)	0.031*	53 (29)	49 (48)	0.002*
90-day mortality	0	1 (1.7)	0.293	7 (2.6)	3 (2.9)	1.000	2 (1.1)	8 (7.8)	0.005*
Major morbidity (Clavien–Dindo grade > II)	5 (3.6)	3 (5.1)	0.603	28 (10)	10 (9.5)	0.782	26 (14)	19 (18)	0.335
All complications	11 (7.9)	8 (14)	0.197	52 (19)	20 (19)	0.925	45 (24)	30 (29)	0.388
Postoperative liver failure	1 (0.7)	4 (6.9)	0.027*	11 (4.1)	7 (6.7)	0.295	11 (6.0)	20 (19)	0.001*
Ascites	0	2 (3.5)	0.085	5 (1.9)	4 (3.8)	0.277	3 (1.6)	3 (2.9)	1.000
Bile leakage	1 (0.7)	0	1.000	6 (2.3)	3 (2.9)	0.716	6 (3.3)	0	0.091
Surgical site infection	5 (3.5)	1 (1.7)	0.673	8 (3.0)	2 (1.9)	1.000	4 (2.1)	0	0.300
Pneumonia	2 (1.4)	0	1.000	7 (2.6)	3 (2.8)	1.000	13 (7.0)	5 (4.8)	0.614
Ileus	0	0	1.000	4 (1.5)	1 (1.0)	1.000	1 (0.5)	0	1.000
Gastrointestinal ulcer	1 (0.7)	0	1.000	3 (1.1)	0	0.562	2 (1.1)	0	0.538
Others (renal/heart failure, etc.)	1	1		8	0		5	2	
Postoperative stay, days	6 (5–8)	6 (5–8)	0.958	8 (7–11)	7 (5–9)	< 0.001*	9 (7–12)	8 (6–11)	0.038*
Survival outcome									
RFS			0.117			0.768			0.906
1-year RFS, %	80	73		78	74		54	58	
3-year RFS, %	61	43		52	50		32	29	
5-year RFS, %	48	43		38	40		29	26	
OS			0.003*			0.985			0.002*
1-year OS, %	98	91		96	92		88	79	
3-year OS, %	93	76		81	80		68	52	
5-year OS, %	84	59		69	69		51	32	

Data are presented as *n*, *n* (%), or median (interquartile range)

JIS, Japan Integrated Staging; CSPH, clinically significant portal hypertension; OS, overall survival; RFS, recurrence-free survival

*Statistically significant

**Fig. 1:**
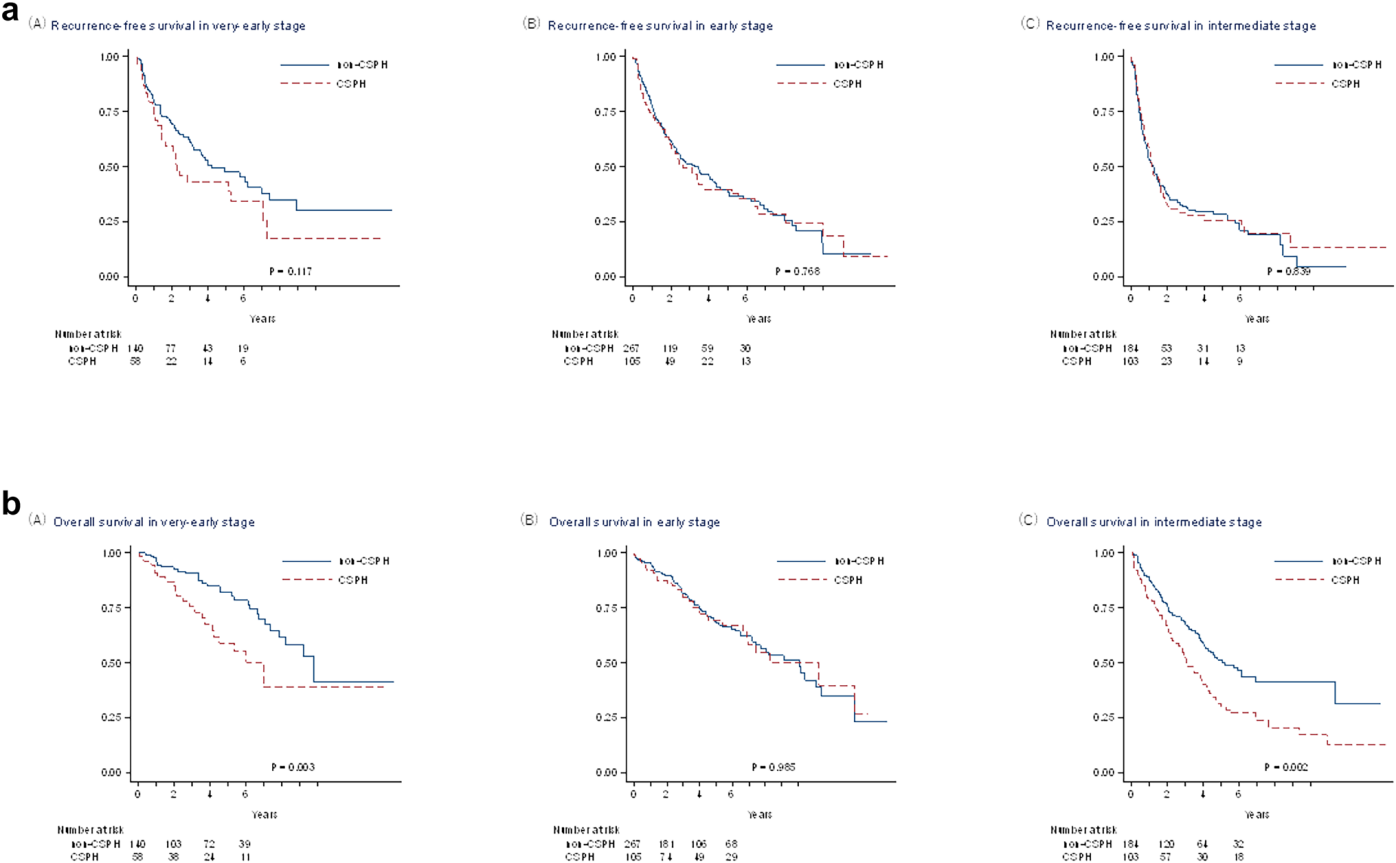
**a** Recurrence-free survival and **b** overall survival in patients with and without clinically significant portal hypertension (CSPH), stratified by Japan Integrated Staging (JIS) score: (A) very-early stage (JIS-score 0), (B) early stage (JIS-score 1), and (C) intermediate stage (JIS-score 2–4)

Kaplan–Meier plots (Fig. 1a for RFS, Fig. 1b for OS) showed no significant differences in RFS between CSPH and non-CSPH groups at any stage. However, OS was significantly lower in CSPH patients in very-early stage (1-, 3-, 5-year OS: 91%, 76%, 59% vs. 98%, 93%, 84%; *p* = 0.003) and intermediate-stage HCC (79%, 52%, 32% vs. 88%, 68%, 51%; *p* = 0.002), but similar in early stage disease (*p* = 0.985).

Table 4 summarizes the results of the univariate and multivariate analyses in the Cox regression model. In univariate analysis, CSPH was associated with worse OS in very-early (HR 2.10 [1.21–3.64], *p* = 0.008) and intermediate-stage HCC (HR 1.69 [1.22–2.38], *p* = 0.002), but not in early stage HCC (HR 1.00 [0.67–1.51], *p* = 0.985). In the multivariate analysis, independent prognostic factors for OS in very-early stage HCC included age (> 70 years), differentiation, and CSPH (HR 2.18 [1.14–4.18], *p* = 0.019). In early stage HCC, significant prognostic factors were age (> 70 years), male sex, ALBI grade (≥ 2), tumor size, and cirrhosis, but not CSPH (HR 0.67 [0.40–1.11], *p* = 0.119). For intermediate-stage HCC, ALBI grade (≥ 2), tumor size, R1 resection, and CSPH (HR 1.67 [1.10–2.56], *p* = 0.017) were significant predictors. For RFS, CSPH was not an independent risk factor at any stage (Supplementary Table S1).

**Table 4:** Univariate and multivariate analyses of overall survival according to JIS-score

Variables	Very-early stage (JIS-score 0) [*n* = 198]						Early stage (JIS-score 1) [*n* = 372]						Intermediate stage (JIS-score 2–4) [*n* = 287]					
	Univariate analysis			Multivariate analysis			Univariate analysis			Multivariate analysis			Univariate analysis			Multivariate analysis		
	HR	95% CI	*p*	HR	95% CI	*p*	HR	95% CI	*p*	HR	95% CI	*p*	HR	95% CI	*p*	HR	95% CI	*p*
Age, years																		
≤ 70	Reference			Reference			Reference			Reference			Reference			Reference		
> 70	1.55	0.89–2.69	0.119	1.85	1.01–3.39	0.046*	1.52	1.03–2.24	0.034*	1.64	1.09–2.46	0.017*	1.11	0.80–1.54	0.540	1.10	0.78–1.55	0.596
Sex																		
Male	Reference						Reference			Reference			Reference			Reference		
Female	0.92	0.50–1.72	0.803				0.77	0.49–1.22	0.272	0.55	0.34–0.91	0.019*	1.14	0.77–1.69	0.509	1.10	0.73–1.67	0.638
Metabolic syndrome																		
No	Reference						Reference			Reference			Reference			Reference		
Yes	0.74	0.43–1.27	0.288				0.88	0.58–1.34	0.560	0.64	0.42–1.04	0.070	0.79	0.55–1.12	0.186	0.86	0.59–1.27	0.456
Child–Pugh grade																		
A	NA						Reference			Reference			Reference			Reference		
B	NA	NA	NA				1.18	0.62–2.27	0.613	1.28	0.57–2.86	0.551	1.27	0.91–1.79	0.160	0.73	0.45–1.18	0.196
ALBI score																		
Grade 1	Reference			Reference			Reference			Reference			Reference			Reference		
Grade 2/3	1.58	0.91–2.75	0.107	1.52	0.84–2.75	0.164	1.76	1.21–2.68	0.003*	1.68	1.10–2.57	0.017*	1.79	1.28–2.51	0.001*	1.77	1.17–2.68	0.007*
Tumor size, cm																		
	0.80	0.41–1.57	0.513	0.77	0.37–1.61	0.489	1.07	1.01–1.15	0.035*	1.10	1.01–1.20	0.022*	1.03	0.99–1.07	0.116	1.06	1.00–1.11	0.032*
Tumor number																		
Single nodule	NA						Reference			Reference			Reference			Reference		
Multiple nodules (≥ 2)	NA	NA	NA				0.84	0.50–1.41	0.508	0.89	0.43–1.85	0.751	0.90	0.69–1.19	0.472	1.04	0.64–1.69	0.873
Differentiation																		
Well/moderate	Reference			Reference			Reference			Reference			Reference			Reference		
Poor/other	4.13	1.59–10.7	0.004*	3.10	1.15–8.31	0.025*	1.34	0.68–2.67	0.397	1.45	0.72–2.93	0.300	1.22	0.80–1.85	0.358	0.94	0.60–1.49	0.805
Microvascular invasion																		
No	NA						Reference			Reference			Reference			Reference		
Yes	NA	NA	NA				1.60	0.78–3.31	0.202	1.94	0.87–4.31	0.103	1.11	0.80–1.55	0.528	1.04	0.66–1.66	0.857
Cirrhosis																		
No	Reference			Reference			Reference			Reference			Reference			Reference		
Yes	1.26	0.70–2.27	0.439	0.94	0.45–1.94	0.857	1.48	1.01–2.18	0.046*	1.89	1.19–3.02	0.007*	1.36	0.97–1.91	0.079	1.20	0.77–1.88	0.413
Resection type																		
Non-anatomical	Reference						Reference			Reference			Reference			Reference		
Anatomical	0.50	0.18–1.39	0.183				1.14	0.78–1.66	0.504	1.09	0.69–1.72	0.723	1.02	0.74–1.41	0.894	1.29	0.80–2.08	0.302
Margin status																		
R0 resection	Reference			Reference			Reference			Reference			Reference			Reference		
R1 resection	1.63	0.79–3.35	0.185	1.46	0.70–3.05	0.314	1.65	0.83–3.28	0.151	1.71	0.82–3.56	0.152	1.94	1.21–3.08	0.005*	1.83	1.12–2.97	0.015*
Liver resection																		
Open approach	Reference			Reference			Reference			Reference			Reference			Reference		
Minimum invasive approach	0.96	0.55–1.69	0.899	0.99	0.54–1.82	0.987	1.05	0.71–1.55	0.815	1.14	0.69–1.87	0.616	1.09	0.78–1.53	0.615	1.18	0.74–1.87	0.491
CSPH																		
No	Reference			Reference			Reference			Reference			Reference			Reference		
Yes	2.10	1.21–3.64	0.008*	2.18	1.14–4.18	0.019*	1.00	0.67–1.51	0.985	0.67	0.40–1.11	0.119	1.69	1.22–2.38	0.002*	1.67	1.10–2.56	0.017*

JIS, Japan Integrated Staging; CSPH, clinically significant portal hypertension; HR, hazard ratio; NA, not applicable

*Statistically significant

Table 5 shows postoperative recurrence, additional treatments, and causes of death. Residual liver recurrence (including intrahepatic and local recurrence) was the most frequent recurrence pattern across all stages, with similar post-recurrence treatment strategies between CSPH and non-CSPH groups. HCC recurrence was the main cause of death in all groups. Liver decompensation-related mortality was similar between CSPH and non-CSPH groups in early stage HCC (12% vs. 15%, *p* = 0.758) but significantly higher in CSPH patients in very-early (3.3% vs. 18%, *p* = 0.027) and intermediate stages (9.0% vs. 34%, *p* < 0.001).

**Table 5:** Postoperative recurrence patterns, details of additional treatment after recurrence, and causes of death

	JIS-score 0(n = 93)		*p*	JIS-score 1 (n = 192)		*p*	JIS-score 2–4 (n = 192)		*p*
	Non-CSPH (*n* = 63)	CSPH (*n* = 30)		Non-CSPH (*n* = 135)	CSPH (*n* = 57)		Non-CSPH (*n* = 125)	CSPH (*n* = 67)	
Recurrence site			0.174			0.785			0.121
Local recurrence in the liver	3 (4.7)	5 (17)		15 (11)	8 (14)		8 (6.4)	4 (6.0)	
Intrahepatic	58 (91)	25 (83)		114 (84)	48 (84)		105 (84)	62 (93)	
Peritoneal dissemination	1 (1.6)	0		1 (0.7)	0		3 (2.4)	0	
Bone	2 (3.1)	0		1 (0.7)	1 (1.8)		1 (0.8)	1 (1.5)	
Lung	0	0		3 (2.2)	0		8 (6.4)	0	
Lymph node	0	0		1 (0.7)	0		0	0	
Retreatment after recurrence	55	25	0.750	109	51	0.749	107	52	0.167
1 procedure	34 (62)	16 (64)		63 (58)	27 (53)		59 (55)	26 (50)	
Re-resection	15	7		22	9		14	6	
RFA	4	1		7	4		1	2	
TACE	12	6		21	11		22	15	
Chemotherapy	2	1		11	1		17	1	
CyberKnife	1	1		2	2		5	2	
2 procedures	16 (29)	6 (24)		34 (31)	19 (37)		36 (34)	23 (44)	
Re-resection + RFA	1	0		1	0		1	0	
Re-resection + TACE	4	0		6	2		3	6	
Re-resection + chemotherapy	1	1		2	0		4	0	
Re-resection + CyberKnife	1	0		1	1		1	0	
RFA + TACE	3	1		7	4		4	3	
RFA + chemotherapy	2	0		1	0		2	0	
RFA + CyberKnife	0	0		2	0		1	0	
TACE + chemotherapy	3	3		14	9		18	13	
TACE + CyberKnife	0	1		0	3		0	1	
Chemotherapy + CyberKnife	1	0		0	0		2	0	
More than three procedures	5 (9.0)	3 (12)		12 (11)	5 (9.8)		12 (11)	3 (5.8)	

	JIS-score 0 (*n* = 52)			JIS-score 1 (*n* = 107)			JIS-score 2–4 (*n* = 101)		
	Non-CSPH (*n* = 30)	CSPH (*n* = 22)		Non-CSPH (*n* = 74)	CSPH (*n* = 33)		Non-CSPH (*n* = 78)	CSPH (*n* = 68)	
HCC recurrence	18 (60)	9 (41)		43 (58)	23 (70)		56 (72)	38 (56)	
Hepatic decompensation	0	4 (18)		9 (12)	5 (15)		7 (9.0)	23 (34)	
Pneumonia	4 (13)	3 (14)		4 (5.4)	2 (6.1)		2 (2.6)	1 (1.5)	
Cerebrovascular disease	2 (6.8)	0		3 (4.1)	0		3 (3.9)	1 (1.5)	
Leukemia	1 (3.3)	2 (9.1)		0	0		1 (1.3)	0	
Cardiovascular disease	0	1 (4.6)		3 (4.1)	1 (3.0)		1 (1.3)	0	
Other cancer	2 (6.8)	2 (9.1)		6 (8.1)	1 (3.0)		4 (5.1)	3 (4.4)	
Sepsis	0	1 (4.6)		3 (4.1)	0		3 (3.9)	0	
Renal failure	1 (3.3)	0		1 (1.4)	0		0	1 (1.5)	
Gastrointestinal bleeding	0	0		1 (1.4)	0		0	0	
Unknown	2 (6.7)	0		1 (1.4)	1 (3.0)		1 (1.3)	1 (1.5)	

JIS, Japan Integrated Staging; CSPH, clinically significant portal hypertension; RFA, radiofrequency ablation; TACE, transarterial chemoembolization; HCC, hepatocellular carcinoma

*Statistically significant

## Discussion

LR is considered a viable treatment option for patients with HCC and CSPH [6], but its impact on survival remains controversial. Previous studies have suggested that LR can achieve acceptable outcomes in carefully selected patients with CSPH [21, 22]; however, most did not stratify patients based on tumor burden and liver function in detail. To address this limitation, we categorized patients using the JIS-score into three groups: very-early stage (JIS 0), early stage (JIS 1), and intermediate stage (JIS 2–4). This stratification revealed that CSPH was significantly associated with poorer survival in the very-early and intermediate-stage groups, whereas early stage HCC patients with CSPH had comparable outcomes to those without CSPH. These findings suggest that patients who have early stage HCC with CSPH may be suitable candidates for LR, while those with very-early or intermediate stages require more cautious consideration.

Several studies comparing LR outcomes in CSPH and non-CSPH patients have generally reported worse outcomes in the CSPH group [7, 8]. Bruix et al. [2] concluded that CSPH is a risk factor for postoperative liver dysfunction and worse 5-year survival even with preserved liver function. However, recent studies have shown that CSPH does not affect the prognosis of patients with early stage HCC [9, 10]. For instance, a prospective study found that laparoscopic LR in patients who had BCLC stage 0 or A with CSPH was safe and provided outcomes comparable to those of patients without CSPH [23]. These conflicting conclusions may arise from differences in patient demographics, small sample sizes, and insufficient adjustment for confounding factors. Two meta-analyses found that CSPH significantly affected OS after LR but also reported high heterogeneity among studies [3, 24]. Given this controversy, our study aimed to clarify the stage-specific impact of CSPH on surgical outcomes. Using one of the largest sample sizes to date, we stratified patients with HCC by JIS-score while adjusting for potential confounders. These methodological strengths enhance the robustness of our findings and provide valuable insights for future research.

Interestingly, CSPH had a less pronounced impact on OS in early stage HCC, which may be due to several factors. First, oncological factors appeared to be more favorable in the CSPH group. The JIS-score is determined by tumor size and Child–Pugh grade [12], and although Child–Pugh B was more common in patients with CSPH (24% vs. 2.2%, *p* < 0.001), tumors were smaller in diameter (2.3 vs. 3.2 cm, *p* < 0.001). Because of this oncological factor, non-anatomical resection and MIS were more frequently performed in the CSPH group, resulting in shorter operative durations and less blood loss. In patients with CSPH who are more likely to be eligible for MIS, a less-invasive procedure may have influenced short-term outcomes. Previous studies have reported that MIS is associated with better short-term outcomes, potentially contributing to comparable 90-day mortality rates and postoperative liver failure incidence between the CSPH and non-CSPH groups, despite patients with CSPH having worse liver function (Child–Pugh grade, MELD score, and ALBI score) [23, 25]. In this study, a subgroup analysis of open versus MIS in patients with CSPH also showed significantly better short-term outcomes with MIS (Supplementary Table S2). Second, unlike the very-early stage, where the T factor is fixed at 1, patients with early and intermediate-stage HCC include a broader range of tumor sizes and liver function statuses. This heterogeneity may explain why the negative prognostic impact of CSPH appeared diluted in the early stage group, in contrast to the very-early and intermediate stages, where liver function tends to be either consistently preserved or consistently impaired. Notably, 24% of patients with early stage CSPH were classified as Child–Pugh B and 52% were ALBI grade 2, compared with 65% and 80% in the intermediate-stage CSPH group, respectively. Although ALBI grades were significantly worse in the CSPH group than in the non-CSPH group for both early and intermediate stages, we accounted for these differences in our multivariate analysis. These findings suggest that within the same prognostic group, LR for early stage HCC may attenuate the adverse impact of CSPH—an effect not observed in intermediate-stage HCC. Additionally, despite similar post-recurrence treatments across all stages, deaths due to postoperative liver decompensation were not significantly increased in patients with early stage CSPH. Careful patient selection and surgical approach in early stage HCC may allow LR to balance surgical and hepatic risks, reducing the prognostic impact of CSPH. Conversely, patients with very-early stage HCC generally have preserved liver function (Child–Pugh and ALBI grades) and small tumors, leading to similar surgical approaches in both CSPH and non-CSPH groups. However, postoperative liver failure was more frequent in patients with CSPH, and mortality related to liver decompensation was significantly higher. In very-early stage HCC, non-anatomical and minimally invasive resections—surgical approaches associated with a lower physiological burden—were performed equally in both the CSPH and non-CSPH groups. However, these approaches may not have been sufficient to offset the adverse effects of portal hypertension on liver function in the CSPH group. Therefore, although short-term outcomes did not differ significantly, liver failure—primarily Grade A—appeared to be more frequent in the CSPH group. Additionally, patients with CSPH tended to have a higher prevalence of cirrhosis, although this difference was not statistically significant. We believe that the increased mortality in the CSPH group likely reflects the broader negative impact of CSPH on the natural progression of cirrhosis. In intermediate-stage HCC, as in early stage HCC, non-anatomical and minimally invasive approaches were more commonly used in the CSPH group. In the early and intermediate stages, wound infections and pneumonia—less severe complications that can prolong hospital stays—were more common in the non-CSPH group. Interestingly, despite higher rates of major complications and liver failure in patients with CSPH, those in the intermediate stage had shorter hospital stays. This paradox may be explained by the more frequent use of minimally invasive surgery in patients with CSPH in both the early and intermediate stages, as minimally invasive surgery is known to shorten hospital stays following LR. Nevertheless, short-term outcomes, including postoperative liver failure and 90-day mortality, were worse in the CSPH group, and more patients died of liver decompensation in the long term. These findings suggest that in intermediate-stage HCC, the impact of LR on residual liver function is greater in patients with CSPH, making it challenging to balance oncological benefits with hepatic risks. Future research is needed to develop new criteria for surgery in patients who have intermediate-stage HCC with CSPH, ensuring that good outcomes can be expected.

To further validate the impact of CSPH, we analyzed postoperative OS stratified by the BCLC staging system (very-early: BCLC-0; early: BCLC-A; intermediate: BCLC-B) [6]. The results were consistent with those from the JIS-score analysis (Supplementary Tables S3–5), confirming that CSPH was an independent risk factor for OS in very-early and intermediate-stage patients but not in early stage patients, reinforcing the minimal impact of CSPH on OS in early stage HCC. The JIS-score has been reported to be superior to BCLC staging in predicting prognosis, because BCLC staging encompasses a broad spectrum of patients within each category, particularly in the early stage group (BCLC-A) [11]. Therefore, JIS scoring may provide a more precise assessment of CSPH’s impact on LR outcomes, especially in early stage HCC.

This study has several limitations. First, as a retrospective study, selection and confounding biases are inevitable, even with multivariate adjustments. Future multicenter prospective studies with standardized CSPH diagnostic criteria are needed to validate our findings. Second, while CSPH can be diagnosed using non-invasive criteria, the invasive measurement of HVPG (≥ 10 mmHg) is considered the gold standard for detecting portal hypertension [26]. Although HVPG measurement is ideal, its clinical application is challenging; therefore, non-invasive criteria were used in this study [17, 18]. Third, this study was conducted at a single institution, which may limit the generalizability of the findings to other centers. We selected only patients with HCC and CSPH and analyzed risk factors for outcomes within this group (Supplementary Table S6). Our findings indicate that variceal grade is not an independent risk factor for OS, whereas tumor diameter and ALBI grade are significant predictors. However, these factors alone may not be sufficient to establish new surgical selection criteria. Therefore, we believe that future multicenter studies are needed to further refine and validate these criteria. Finally, CSPH was assessed only preoperatively without evaluating postoperative changes, underscoring the need for future studies to assess postoperative CSPH progression.

In conclusion, patients with early stage HCC (JIS 1 or BCLC-A) and CSPH have long-term prognoses comparable to those without CSPH. However, CSPH is associated with poorer survival in patients with very-early and intermediate-stage HCC undergoing LR. Surgery should be actively considered for patients with early stage HCC regardless of CSPH, while a more cautious evaluation is warranted for patients with very-early or intermediate stages. For patients with very-early or intermediate-stage HCC and CSPH, alternative strategies, such as radiofrequency ablation, transarterial chemoembolization with chemotherapy, or liver transplantation, should be carefully considered alongside LR.

## Lay summary

We examined the impact of clinically significant portal hypertension (CSPH) on liver resection outcomes in patients with hepatocellular carcinoma (HCC). A key strength of this study is the detailed stratification using the Japan Integrated Staging score, which clarified the effects of CSPH across different tumor stages, particularly in early stage HCC. Our findings suggest that CSPH does not uniformly contraindicate liver resection; rather, its impact varies based on tumor burden and liver function. While patients who had early stage HCC with CSPH exhibited survival outcomes comparable to those without CSPH, CSPH was associated with worse survival in very-early and intermediate stages. These insights may help refine surgical decision-making for patients who have HCC with CSPH.

## Supplementary Information

Below is the link to the electronic supplementary material.

Supplementary file1 (XLSX 51 kb)

## Data Availability

The data that support the findings of this study are available on request from the corresponding author.
